# Osteopathic Cranial Manipulative Medicine as a Proposed Addition to the Treatment Regimen for Idiopathic Sudden-Onset Unilateral Sensorineural Hearing Loss: A Case Report

**DOI:** 10.7759/cureus.49728

**Published:** 2023-11-30

**Authors:** Reddog E Sina, J'Aimee Lippert, Katherine Guardardo

**Affiliations:** 1 Osteopathic Manipulative Medicine, Michigan State University College of Osteopathic Medicine, East Lansing, USA

**Keywords:** adult, eustachian tube, osteopathic manipulation/methods, sensorineural hearing loss/diagnosis, sensorineural hearing loss/treatment, sudden hearing loss/diagnosis, sudden hearing loss/treatment

## Abstract

Adult sudden-onset unilateral sensorineural hearing loss is primarily idiopathic. There are few treatment options with high success rates, but the literature suggests there is equally moderate success with oral or intratympanic steroids, with some evidence to support the use of acupuncture as a salvage treatment. Here, we present a case of sudden unilateral sensorineural hearing loss with otolaryngology evaluation and audiograms before and after osteopathic manipulative treatment (OMT). Osteopathic cranial manipulative medicine (OCMM) directed at eustachian tube dysfunction elicited findings indicating resolution of symptoms was at least partly due to cranial treatment. This report explains a probable anatomic mechanism contributing to hearing loss and suggests treatment options that should be considered as part of the treatment of idiopathic unilateral hearing loss. Future research opportunities are also proposed.

## Introduction

Adult unilateral sensorineural hearing loss (USNHL) is differentiated from bilateral hearing loss, an often genetic or developmental conductive or mixed conductive-sensorineural condition that predominantly presents in childhood [1]. USNHL is defined as a spontaneous hearing loss of greater than 30 dB over three contiguous frequencies within a 72-hour period, occurring with similar incidence in men and women in all age groups but with the highest occurrence during the fifth and sixth decades [2]. Nevertheless, some studies report a slightly increased likelihood of occurrence in men [3].

All cases of bilateral hearing loss are known to be about 50% genetic, whereas most sudden-onset unilateral hearing loss is idiopathic, although cases have also been attributed to infections (12.8%), otologic disease (4.7%), trauma (4.2%), vascular or hematologic causes (2.8%), neoplastic (2.3%), and other causes (2.2%) [3]. Sudden-onset sensorineural hearing loss (SSNHL) is considered an emergency and calls for a swift diagnosis and treatment [3,4]. In addition to hearing loss, it may also present with blocked ear sensations and vertigo [2]. One study reported concurrent tinnitus in 70%-80% of patients [5]. SSNHL is reported to be up to 71%-90% idiopathic, affecting 5-27 per 100,000 people and about 66,000 new cases in the United States annually [4]. However, the incidence may be greater due to unaccounted cases with prompt recovery who do not seek medical care [3]. In addition to the etiologies above, other known causes include ototoxicity (Ménière’s disease), autoimmune diseases [4,6] or, more rarely, auditory neuropathy, chemoradiotherapy, dialysis, inner ear malformation, acute otitis media, multiple sclerosis, vertebrobasilar dolichoectasia, microcirculation disturbance, systemic autonomic disorders, thyroid disorders, and ruptured cochlear membranes [3,7]. 

Risk factors for idiopathic sudden sensorineural hearing loss (ISSNHL) include cigarette smoking, hypertension, hyperlipidemia, ischemic vascular disease, diabetes mellitus, and hyperfibrinogenemia [3]. There are six categories of ISSNHL: hearing loss in low frequencies within 250-500 Hz (higher prognosis); middle frequencies around 1000 Hz (moderate prognosis); higher frequencies above 4000 Hz (poor prognosis); flat-type hearing loss at all frequencies (very poor prognosis); complete deafness at all frequencies (worst prognosis); and other undetermined prognoses. At least two-thirds of ISSNHL patients recover within two weeks, and after that time, recovery becomes less likely [5].

In 2012, the American Academy of Otolaryngology-Head and Neck Surgery (AAOHNS) published guidelines for managing SSNHL due to the dearth of clear data to guide treatment. This was updated in 2019 [8] using the best-collected research, meta-analyses, and case studies they could collect. Their standard approach became physical examination, including Weber and Rinne testing, possible MRI or auditory brainstem response, steroids (systemic or intratympanic), and possible salvage therapy with hyperbaric oxygen therapy and an increased concentration of intratympanic steroid injections. The AAOHNS advises against antivirals, lab tests, and vasodilators and describes several unsupported “complementary” treatments (based on a lack of evidence), suggesting they may be dangerous, including acupuncture [4,8]. They do not address treatment options with any form of manual medicine.

Acupuncture is generally utilized only as a salvage therapy for ISSNHL after disease onset and observationally. It appears to be advocated more often in studies originating in China, as western physicians outside the U.S. military have been slow to adopt acupuncture as a useful modality for the treatment of many conditions and illnesses other than back pain. It’s thought that acupuncture enhances blood flow to the ear and reduces blood viscosity. In addition, acupuncture at the ear acupoints may enhance the inflammatory response, lymph circulation, and conductivity of the auditory nerve [5,9]. Some studies have shown that acupuncture results in improved hearing in contrast to other Western medicine treatments [9]. Nevertheless, there are some studies stating that acupuncture alone may not be effective in treating ISSNHL [10].

We were unable to locate research documenting the effect of osteopathic manipulative treatment (OMT) in ISSNHL, except for one article describing general applications of osteopathic models of evaluation and treatment to adult hearing loss, which is comprehensive and very useful [11] and consistent with the recommendations below.

In some cases of SSNHL, hearing thresholds may not be recovered. Factors affecting recovery include age, severity, frequencies affected, vertigo, and time between onset and treatment [2]. In this situation, evidence-based audiograms demonstrate rapid and complete resolution of profound temporary hearing loss following the established treatment practice of high-dose steroids, adding in acupuncture as the most common salvage treatment, but the addition of OCMM may provide some insight into additional pathways to follow in treating idiopathic cases.

## Case presentation

A 60-year-old male presented to an Osteopathic Manipulative Medicine (OMM) clinic, reporting that two days earlier, he had awakened with “complete” left-sided hearing loss after sleeping on his left side (which was a normal position). The patient has a known history of bilateral tinnitus, reportedly from noise exposure since he was 16 years old, which has worsened during this same time period. He also reports fullness and an inability to discern language in the left ear. The patient admits to previous loud noise exposure but denies a history of any previous significant hearing issues, COVID-19 exposure, trauma, pain, balance issues, or recent viral or bacterial illness/exposure; furthermore, he denies personal or family history of hearing loss. A review of psychosomatic diagnostics did not reveal findings suggesting a non-organic etiology.

Other history includes treatment for chylomicronemia and a predominantly vegan diet except for cold-water fish, supplemented with vitamin D and additional Omega-3 fatty acids. He exercises regularly, has no other concurrent health issues, and has a BMI in the low-normal range. The patient denies any history of stroke, transient ischemic attacks, or other neurologic changes.

Otolaryngologist and audiologist evaluation

One day after awakening with unilateral hearing loss, the patient was seen by his otolaryngologist for an urgent ear and audiology exam; the visit note reports no abnormalities on examination aside from apparent unilateral idiopathic hearing loss, and the audiologist reports that the patient complained of aural structure “fullness.” An audiogram was performed, which showed similar ear canal volume (R 1.10 cc and L 0.95 cc), suggesting the left was slightly smaller but both at the larger end of normal, suggesting there was no significant blockage of the ear canal; peak admittance (R 0.21 ml vs. 0.25 ml); and curve type (both low compliance); however, the negative peak air pressure on the left was -50 decaPascals (daPa) to -25 daPa, doubling the tympanic membrane's resistance to movement (Table 1).

**Table 1:** A diagnostic tympanogram was performed on day 2.

Day 2: Diagnostic Tympanogram		
Probe Tone (Hz)	Right	Left
Ear Canal Volume (cc)	1.10	0.95
Peak Admittance (ml)	0.21	0.25
Peak pressure (daPa)	-25	-50
Curve type	As (low compliance)	As (low compliance)

The tympanogram demonstrated left-sided hearing levels significantly lower than those measured on the right. The audiologist's interpretation suggests normal right-sided hearing to 3 kilohertz (kHz), decreasing in the 4-8 kHz range and being evaluated as moderate to moderately severe sensorineural hearing loss. The left side demonstrated mild hearing loss at 0.25 kHz, moderately severe to severe in the 0.5-3 kHz range, and improved to moderate sensorineural hearing loss in the 4-8kHz range (Table 2).

**Table 2:** Hearing level in decibels (dB) from the tympanogram performed on day 2.

Frequency in Hertz (Hz)	Right Ear	Left Ear
250	10 (unmasked)	Air 40 (masked) Bone 30 (masked)
500	5 (unmasked)	Air 70 (masked) Bone 60 (masked)
750	5 (unmasked)	Air 80 (masked)
1000	10 (unmasked)	Air 80 (masked)
1500	15 (unmasked)	Air 80 (masked) Bone 75 (masked)
2000	15 (unmasked)	Air 75 (masked) Bone 70 (masked)
3000	20 (unmasked)	Air 70 (masked)
4000	45 (unmasked)	Air 55 (unmasked) Bone 50 (masked)
6000	70 (unmasked)	Air 55 (unmasked)
8000	75 (unmasked)	Air 50 (unmasked)

The results of physical examination and frequency-specific testing underscored the larger picture of hearing loss: even with assistance, there was a profound loss of air and bone conduction, speech reception, and word recognition on the left side. The patient's pure tone average by air conduction (AC) on the right was 10 dB hearing loss (dBHL), while on the left, it was 75 dBHL and 66 dBHL for bone conduction (BC). The speech reception/awareness threshold on the right was also 10 dB for AC, but on the left, it was 70 dB. Word recognition on the right was 100% at 55 dB, and on the left was 28% at 85 dB and 90 dB. Peak frequency loss was between 4 and 8 kHz (Table 3).

**Table 3:** Day 2: Pure tone average, speech reception, and word recognition

Test		Right Ear	Left Ear
Pure Tone Average	Monaural	Air 10 dBHL [3a]	Air 75 dBHL [3a]
	Soundfield	unaided	aided
Speech reception/Awareness Threshold		Air 10 dB [SRT]	Air *70 dB [SRT]
Word Recognition		100% at 55 dB	*28% at 85 dB *28% at 90 dB

The patient reports that the otolaryngologist's conclusion suggested that if the patient's hearing did not return within three weeks, there was a good chance of permanent profound hearing loss, consistent with the literature. The patient reported he was offered intratympanic or oral steroids and opted for 50 mg/day oral prednisone, but no imaging or other treatment was offered at that time. 

Therapeutic intervention

The patient was evaluated and treated by the same osteopathic physician (D.O.), a board-certified specialist in osteopathic neuromusculoskeletal medicine using osteopathic cranial manipulative medicine (OCMM). Evaluation and treatment were performed using a predominantly fascial approach with significant attention to anatomy on four occasions, starting two days after the onset of hearing loss. He also received medical acupuncture three times from a formally trained medical professional. The first acupuncture treatment occurred earlier, on the second day of symptoms before arrival at the OMM Clinic. The acupuncturist also treated the patient on days 6 and 9. At no point did he experience any vestibular symptoms or changes in his levels of pre-existing tinnitus.

At the patient's presentation on day two of symptoms, the initial examination confirmed gross left-sided hearing loss to speech and sound. He was placed in a supine position and evaluated for fascial restrictions and impaired cranial bone motion. Cranial findings indicated significant left-sided fascial restrictions that were centered around significant unilateral left-sided temporal bone dysfunction. The temporal bone preferred internal rotation, and the parietal bone restricted its motion at the parietal pivot and at the nexus of the anterior petrous portion with the sphenoid at the petrosphenoid ligament. There was a compression of the occipitomastoid suture and significant restriction of the sphenobasilar synchrondrosis, including an inferior sphenoid shear, with decreased motion of the sphenoid’s left greater wing in comparison to the right and restriction in occipital extension. The left maxilla also demonstrated a mild preference for internal rotation. Initial treatment returned significant motion to the temporal bone and surrounding structures, but there was no immediate change in hearing. The medial and lateral pterygoid muscles were not treated because the patient reported that the acupuncturist had already treated them.

The patient was seen at his home on day four of his symptoms. At that time, he again demonstrated diffuse fascial restriction on the left side of his head but no longer showed evidence of the petrojugular restriction or the sphenoid shear. He continued to have internal restrictions of his left temporal, parietal, and occipital bones and hypertonicity of his left masseter muscle, medial pterygoid (MP) muscle, and submandibular fascia. Within minutes of treatment, he noted hearing crackling in his left ear and, shortly after that, started getting a mild hearing restoration.

On day six of symptoms, the patient reported a partial return of left unilateral hearing that had continued to improve after being treated on day 4. Over the weekend, he experienced some popping sounds while moving his jaw. He had a slow improvement in his hearing, from complete hearing loss six days before, then garbled, faint, and tinny, to finally being able to discern birds chirping on day five. On physical examination, the left temporal bone was again internally rotated with restrictions at the parietal pivot and the sphenoid but improved. The left sphenoid showed decreased flexion but moved better than before, and the occiput was restricted in cranial flexion. The subdermal fascial layer inferior to the left occiput was restricted, but the left occipital-mastoid suture restriction had resolved. Overall, there was a significant improvement in left-sided fascial restriction, especially after treatment that day.

On day nine, after symptom onset, the patient returned and reported improved left unilateral hearing but still had a fullness sensation. The day before this appointment, he had a follow-up audiogram, at which he reported a subjective 70% improvement in hearing. The tympanogram performed on day eight provided data supporting the patient's report. His ear canal volume now measured 1.18 cc on the right and 1.12 cc on the left. According to the audiologist's interpretation, the right ear demonstrated mild to moderate sensorineural hearing loss from 750-1500 Hz, normal levels from 2-3 kHz, and mild hearing loss from 4-8 kHz. The audiologist concluded the patient's hearing was asymmetrical, with the left side worse in the midrange frequencies but better than the right above 6 kHz (Table 4). 

**Table 4:** Hearing level in dB from a tympanogram performed on day 9

Frequency in Hertz (Hz)	Right Ear	Left Ear
250	15 (unmasked)	Air 15 (masked)
500	10 (unmasked)	Air 15 (masked) Bone 20 (masked)
750	(not tested)	Air 45 (masked) Bone 40 (masked)
1000	10 (unmasked)	Air 40 (masked)
1500	(not tested)	Air 40 (masked) Bone 75 (masked)
2000	15 (unmasked)	Air 20 (unmasked)
3000	20 (unmasked)	Air 25 (unmasked)
4000	45 (unmasked)	Air 30 (unmasked)
6000	70 (unmasked)	Air 35 (unmasked)
8000	75 (unmasked)	Air 45 (unmasked)

As compared to the examination from seven days earlier, the patient's pure tone average increased by 48 dBHL (AC) and 50 dBHL (BC). Similarly, the speech reception threshold improved by 45 dB, and word recognition improved to 100% at 65 dB, up from 28% at 85-90 dB. Overall, his gross hearing was nearly equivalent between the right and left sides (Table 5). 

**Table 5:** Day 9: Pure tone average, speech reception threshold, and word recognition

Test		Right Ear	Left Ear
Pure Tone Average	Monaural	Air 12 dBHL	Air 27 dBHL, Bone 25 dBHL
	Soundfield	unaided	aided
Speech Reception Threshold		Air 10 dB	Air *25 dB
Word Recognition		100% at 55 dB	100% at 65 dB

On a physical exam, he demonstrated continued restrictions in left temporal bone motion at the parietal pivot and the sphenosquamous pivot with a recurrence of the inferior sphenoid shear, limiting flexion of the sphenoid and extension of the occiput. Sub-mandibular fascial hypertonicity was present but not as severe as previously.

The patient presented on day 15 post-symptom onset for a recheck with a report that he had received no further acupuncture treatments, had completed his taper of steroids, and that his hearing was subjectively back to baseline. He added that his otolaryngologist was somewhat surprised by his recovery. He indicated that he is under a significant amount of stress and acknowledged that he may be grinding his teeth in his sleep.

## Discussion

Conventional treatment of ISSNHL has traditionally (and justifiably) viewed the problem through the lens of otolaryngology. The orientation, therefore, tends to be pathophysiological, and when there is imaging, such as an MRI, the evaluation of structure is a search for something foreign or visible, with a concern for possible surgery. An osteopathic neuromusculoskeletal perspective analyzes things differently. Based on more than a century’s practice of examining patients according to anatomical structure and function, an osteopathic physician is trained to ask if there is an underlying structural change that has led to a change in function and if there is a way that this change can be addressed so that the body can correct itself. This has been well documented for decades in treating other dysfunctions of the ear, most notably various forms of otitis media, resulting in increased speed of recovery, decreased use of antibiotics, and decreased pain and recurrence [12]. These treatments also focus on the temporal bone, where the vestibulocochlear anatomy resides, and the eustachian tubes. Otolaryngologists use the tools at hand, such as intratympanic or oral steroids [8], diagnostic MRI, ABMs, audiograms, etc., to attempt to address the symptoms, but they are not necessarily seeing the issue, which is a functional motion loss of the affecting delicate anatomy. 

In this case, hearing loss presented unilaterally without facial paralysis or pain and without dizziness, vertigo, or balance issues. Long-term preexisting tinnitus was subjectively increased but was not a focal concern. No trauma was reported, and no history was consistent with any known organic causes of USSNHL. The presentation is therefore not consistent with infectious, traumatic, autoimmune, or psychogenic causes; because this was not part of a larger constellation of bilateral neurological symptoms, it is unlikely to have been caused by a central cerebral lesion or disease process, making an MRI not immediately necessary. However, it would be appropriate if conditions remained unchanged after an extended period without symptomatic relief. 

A review of the anatomy around the internal ear structures may provide some insight into the origin of this condition. Neurologically, the vestibulocochlear nerve (CN VIII) emerges from the brainstem between the pons and medulla oblongata posterior to the facial nerve (CN VII) [13]. The pia mater independently sheathes the nerves; then, the bundle is sheathed by the arachnoid mater and, ultimately, by the periosteum. CN VIII then travels laterally, anteriorly, and superiorly across the petrous portion of the temporal bone to enter the internal acoustic meatus [13].

A speculative explanation for ISSNHL would emerge from following the anatomic path of the vestibulocochlear nerve as it travels from the brainstem, then splitting off when the vestibular nerve enters the vestibule, and a short section (about 2 mm) of the cochlear nerve passes over an area of the temporal bone before entering the cochlea [14]. In this scenario, the cochlear branch is exposed to the temporal bone and possibly inhibited if the temporal bone’s motion is altered. This hypothesis would require a functional MRI (fMRI) to confirm, and since the length of the nerve that would have to be affected is so short, this scenario seems highly improbable. A more straightforward and likely repeatable suggestion is based on the osteopathic concept of the relationship between structure and function as it relates to the anatomy around the eustachian tube (ET). The ET is surrounded by the tensor veli palatini (TVP) and the levator veli palatini (LVP) proximally within the temporal bone. Medial pterygoid (MP) and lateral pterygoid (LP) tone control the ET's lumen diameter distally, affecting its capacity to allow fluid and air exchange [15,16].

Three muscles are thought to affect the function of the ET. The TVP originates at the sphenoid spine, scaphoid fossa, lateral lumina of the tubal cartilage, posterior half of the membranous tubal wall, and the salpingopharyngeal fascia. Increasing the TVP’s tension pulls laterally on the exterior wall of the ET, opening its lumen. The levator veli palatini (LVT) does not touch the ET, but it has connections to the fascia around it, and changes in the LVT’s tension are thought to contribute to the shape of its lumen [13]. The medial pterygoid (MP) closes the jaw and contributes to protruding the mandible. If the TVP tone is increased, such as by opening the mouth, its fibromuscular attachments to the Weber-Liel fascia, which is also attached to the TVP by the fibromuscular fascia, mechanically relax the MP and expand the ET’s lumen. Closing the mouth will do the opposite, relaxing the TVP, engaging the MP, and releasing the lateral tension on the eustachian tube, closing its lumen [14,16]. This causes a pumping motion that helps to clear the ears during the process of opening and closing the jaw by allowing either air or fluid exchange. Failure of this mechanism due to muscle hypertonicity or bony motion restrictions results in a failure of this pumping mechanism and results in either increased unilateral intraotic air or fluid pressure.

Concurrent restriction of temporal and sphenoid bone motion from surrounding structures (such as the parietal bone, occiput, and facial bones) complicates the release of the muscles surrounding the ET [16]. It severely restricts its capacity to cyclically open and close, clearing the middle ear and allowing for normalized fluid and airflow to and from the middle ear. The application of OCMM to the muscles, especially the MP, and the structures to which these muscles are connected-the temporal bone, parietal bone, zygoma, sphenoid, occiput, jaw, and maxilla-are ways to access the ET and restart the pumping mechanism that can normalize fluid and air pressure in the middle ear, allowing hearing to return [17].

This suggests the mechanism of injury, in this case, is likely pressure on the jaw or bruxism during sleep (which the patient eventually admitted he experienced). Grinding creates sustained pressure on the ET, keeping its lumen closed and allowing pressure to build until it causes an air pressure buildup that can affect hearing. 

In this case, beginning with the second visit, the patient reported hearing some crackling sounds and a gradual return of his hearing, suggesting that the constant pressure on his ET was alleviating and hearing was returning as cyclic airflow and pressure were normalizing. A similar situation arises when changing altitudes on an airplane, which is assisted by chewing gum to use the TVP and MP to help the ET equilibrate middle ear pressure. There are examples in the osteopathic literature of using cranial techniques to treat fascial, osseous, and myofascial restrictions that affect hearing, including techniques to stretch the fascia connected to the LVP, creating a pump that opens the ET [16,18]. Osteopathic textbooks have been advocating this for nearly one hundred years, and similar treatments have been standard in the teaching of OCMM.

## Conclusions

This is an area that calls for more research. If SSNHL was a common occurrence, large studies could be performed comparing standard treatment with standard treatment plus OCMM. An alternative would be to build a protocol that could be performed by any otolaryngologist and then track results with the added treatment. Audiology studies using tympanometry or other methods to observe changes in middle ear pressure by manipulating the ET, cranial bones, or intraoral muscles would be insightful, but the authors did not locate this type of research during their literature search. The Chinese medical literature advocates earlier referral to acupuncture; direct or indirect MP treatment is appropriate and likely to benefit hearing restoration. We argue that these results would be enhanced by improving cranial bone motion. Based on the anatomy and treatment success detailed in this case study, referral for OCMM, especially including intrabuccal treatment to address the MP, LP, TVP, and LVP, may significantly reduce long-term hearing loss from ISSNHL. Furthermore, addressing the underlying issue that may be contributing to the tightening of these muscles and assessing for jaw clenching versus other associated issues (stress, anxiety, pain) would also be appropriate.
